# Shame and Experiences of Psychosis: A Qualitative Inquiry

**DOI:** 10.1111/inm.70070

**Published:** 2025-05-25

**Authors:** Kimberley Davies, Sophie Isobel, Zachary Steel, Sarah Morgan, Julia M. Lappin

**Affiliations:** ^1^ Discipline of Psychiatry and Mental Health School of Clinical Medicine, Faculty of Medicine and Health, UNSW Sydney Kensington New South Wales Australia; ^2^ The Tertiary Referral Service for Psychosis Prince of Wales Hospital Randwick New South Wales Australia; ^3^ Faculty of Medicine and Health The University of Sydney Camperdown New South Wales Australia; ^4^ Early Psychosis Program South Eastern Sydney Local Health District Sydney New South Wales Australia

**Keywords:** psychosis, qualitative, schizophrenia, shame, trauma

## Abstract

Research reports a strong association between the experience of psychosis and shame, but there is a paucity of detailed information on the nature of this relationship. This qualitative study explored the relationship between shame and psychosis from the perspectives of people with lived experience of psychosis. Using a trauma‐informed and shame‐sensitive approach to research, fourteen adults from three mental health services completed semi‐structured face‐to‐face interviews. Reflexive thematic analysis was used to develop four themes: (1) Psychosis can expose underlying shame; (2) Psychosis can cause shame; (3) Efforts to minimise, avoid, and resist shame; and (4) Shame can be triggered in mental health services. The study highlights that psychosis can both contribute to new experiences of shame and reactivate shame from past experiences. Greater consideration of how mental health services can be shame‐sensitive is needed, particularly within efforts to provide trauma‐informed care.

## 1 Introduction

Psychosis refers to a collection of experiences characterised by perceptual changes and a sense of reality that differs from that experienced by others (Arciniegas 2015). This may include sensory hallucinations such as hearing or seeing things that others cannot, delusions (unusual or false beliefs) and difficulty with organising and expressing thoughts (thought disorder) which affects communication and decision‐making (Arciniegas 2015). Research has established that psychotic experiences are widely experienced within the general community, with the majority of these experiences being infrequent and innocuous (Maijer et al. 2018). For some people, however, psychotic experiences can be distressing and disruptive to daily functioning. Psychosis‐related distress can arise from the nature of the experiences themselves (e.g., fears of persecution), or the way in which a person understands and feels about their experiences (Peters et al. 2017). Shame has increasingly been documented as a common correlate of mental distress, including psychosis (Carden et al. 2020). Better understanding of the nature and role of shame and how this may be linked to psychotic experiences may assist in informing approaches to support people living with distressing psychotic experiences.

## 2 Background

Shame is a self‐conscious and distressing emotion arising from an appraisal that one has violated their personal standards or an appraisal that one is being subject to the judgements of others regarding a violation of social standards, provoking feelings of being unworthy, damaged, or bad (Gilbert 2003). Shame may arise from secondary impacts of psychosis, such as alterations to social roles, medication‐related side effects, or restrictive treatment experiences. Additionally, psychotic symptoms may involve shaming content, such as malevolent and derogatory voices (Connor and Birchwood 2013; Corstens and Longden 2013). Shame may also be present due to the emotional impact of past exposure to traumatic or adverse life experiences (La Bash and Papa 2014). Traumatic and adverse life experiences have consistently been found to have increased prevalence in individuals with experience of psychosis than in the general community (Varese et al. 2012).

A 2024 meta‐analysis of 38 studies confirmed a strong association between shame and psychosis, with levels of reported shame found to increase alongside psychosis symptom severity (Davies et al. 2024). Other research has linked the experience of shame to poorer recovery outcomes in individuals who experience psychosis, including a higher likelihood of co‐occurring mental health problems such as depression, anxiety, post‐psychotic trauma (Wood and Irons 2016; Aunjitsakul et al. 2021; Turner et al. 2013) and more frequent suicidal ideation (Davies, Lappin, Isobel, et al. 2025). Higher levels of shame have also been found to be associated with a greater likelihood of experiencing paranoia (Castilho et al. 2019; Davies et al. 2024). Improving understanding of the relationship between psychosis and shame is therefore critical for addressing the negative impacts of psychosis and has implications for care and treatment.

Although shame appears to influence the experience of psychosis, limited research has examined the nature of the experience of shame amongst people living with psychosis. Qualitative research approaches are well‐suited to this task, as they allow for in‐depth exploration of complex, subjective experiences that may be difficult to capture through quantitative approaches. While some existing research has provided insights into related concepts, significant gaps remain regarding how shame is subjectively experienced by individuals with psychosis, the exploration of the context in which shame is experienced and how shame may impact need and preferences for support. The study seeks to address this gap through an exploration of experiences of shame described by people living with psychosis.

## 3 Methods

This qualitative inquiry is part of a wider mixed‐methods study aimed at understanding the role of shame in the relationship between experiences of trauma and psychosis. The qualitative component used an interpretive exploratory approach to explore experiences of shame in people living with psychosis. Such an exploratory and interpretive research framework is particularly appropriate for understanding complex topics and experiences within the context of limited prior research (Stebbins 2001). This approach enabled reflection on how individuals describe and make sense of their experiences, how these experiences contribute to feelings of shame, and how shame influences their perceptions of psychosis, care, and support needs. The study was guided by the research question: *How do people living with psychosis experience and describe shame?* The 21‐item Standards for Reporting Qualitative Research (SRQR) checklist was used to guide study reporting (O'Brien 2014).

### 3.1 Setting

The study was conducted between 2023 and 2024 across three mental health services in Sydney, Australia. The services included an adult community mental health service, an adult mental health rehabilitation inpatient service, and a youth‐focused community‐based early intervention for psychosis service. Institutional ethical approval was received from the South Eastern Sydney Local Health District HREC (Ref: 2022/ETH02000).

### 3.2 Participants

Participants were mental health service users who were: (1) supported by one of the three included mental health services; (2) had a lived experience of psychosis; (3) were aged 18 years or older; and (4) were able to provide consent. Mental health professionals working in the services identified individuals they supported who met criteria and whom they considered the study to be of interest to. Individuals who expressed interest in the study when approached by their clinician and gave permission to be contacted were followed up by KD to discuss study procedures and ask questions. All participants were provided with study information and provided written and verbal consent. Participants were provided with a voucher for taking part in the study in recognition of the time that participation involved. Interviews were scheduled for a future date suitable for the participant. Fourteen individuals participated in the study. Basic demographic data were collected to ensure a diverse sample, including gender, age, cultural background, and length of illness, but are not reported here to support participant confidentiality. As this study interprets and presents participant experiences of shame, additional care and effort were taken to ensure that no individual or circumstance was identifiable in the findings.

### 3.3 Data Collection

Semi‐structured interviews were used to explore participants' experiences in‐depth, whilst providing a consistent topic framework across the interviews (Kallio et al. 2016). This format allows for some structure to the discussion with flexibility for exploring individual perspectives and issues meaningful to participants (Kallio et al. 2016). The approach assisted in creating a conversational setting that promoted rapport between interviewer and participant. Given the sensitive nature of the interviews, a trauma‐informed approach was used in the conduct of interviews (Isobel 2021). This included prioritising participants' sense of safety throughout the process, for instance, inviting participants to arrange the environment as needed, providing a copy of the research questions prior to the interview commencing and validating feelings expressed. The trauma‐informed approach also actively guided the development of interview questions and the study processes, including by ensuring that words used, including the term ‘psychosis’, were acceptable to participants. Where alternate terms were preferred or suggested, these were used throughout interviews. Throughout the study, awareness of the possibility of causing or triggering shame was considered. This was managed as much as possible through prioritising transparency and choice for participants, frequent reflection amongst the research team, and by paying close attention to participant cues throughout and following data collection.

The interview guide explored individuals' thoughts on their identity and personal strengths; reflections on their experiences of psychosis including what they thought contributed to its occurrence, the impact that psychosis experiences have or had on their life; adverse life events and meanings made of these; emotions arising from experiencing psychosis and/or adverse life events, including shame; and experiences accessing mental health services. Whilst the guide provided a general structure for the interviews, the order and depth of topics explored varied according to participant engagement and flow of the conversation. The interview questions were reviewed for language and clarity, and internally tested by both individuals with lived experience of psychosis and clinicians with expertise in supporting individuals with experiences of psychosis (Kallio et al. 2016). Changes to the interview guide were made throughout the study in response to participant feedback and emergent findings, including restructuring the order of questions and adjusting wording.

Interviews were conducted by the first author (KD) on‐site at the service preferred by the participant and ranged between 27 and 77 min (average 48 min). All interviews were audio‐recorded and transcribed. Participants were invited to bring a support person if preferred; however, no participants elected to. Participants were offered a summary of their transcript to provide feedback if they wished. Six requested a transcript summary; however, no changes were suggested, and all confirmed their agreement with the contents. Throughout data collection, the quality of data was continually assessed, and data collection ceased once the data were considered to be rich and incorporating a range of experiences in depth to sufficiently answer the research question (Braun and Clarke 2021).

### 3.4 Data Analysis

Reflexive thematic analysis (TA) was used to analyse the data. The TA was led by the first author, with the support of the supervisory research team who are all clinicians and researchers working in fields of psychosis and/or trauma. TA is a flexible and robust method of analysis developed by Braun and Clarke (2022). TA allowed for exploration of shame within participant experiences, and development of an interpretive shared meaning across experiences (Braun and Clarke 2023). Following the six phases outlined in Braun and Clarke (2022), each transcript was reviewed alongside the recording to familiarise with the data. The transcripts were then re‐read and initial notes and thoughts made on a hard copy. Data were coded in NVivo (Version 12, QSR International Pty Ltd. 2018) in response to the research question. This process was iterative, with previously coded transcripts checked for newly generated codes. Following data extraction from NVivo, iterative categorisation was used to summarise quotes within each code (Neale 2016). This aided deeper engagement with each code and for an initial identification of patterns. Codes were grouped into potential themes. These candidate themes aimed to represent broad concepts in the data, for instance, common responses to parts of the research question, or codes that described similar aspects of experience (e.g., feeling exposed). Candidate themes were reviewed and discussed within the research team and considered alongside the original dataset to check fit. Further refinement was undertaken, for example, some candidate themes that reflected opposing perspectives of the same concept were merged into one, while others were discarded. Potential themes were only discarded if they were determined to not relate to the research question, guided by Braun and Clarke's approach to ‘telling a story that makes sense of’ shame in the context of experiences of psychosis. As the themes were finalised and agreed upon by the research team, summaries were drafted describing the concept that each encompassed and the story to be told. Themes were then expanded on and formally written up, with illustrative quotes to add depth and aid in comprehensively addressing the research question (Lingard 2019).

Reflexive TA is inherently subjective. However, throughout the process, reflexivity was enhanced through regular research meetings and co‐reflection on the analysis process. All researchers read sample transcripts and discussed their understanding of the participants' experiences with explicit acknowledgment of their positioning as clinicians and/or researchers.

## 4 Findings

Throughout the interviews, participants described experiences of shame that were pervasive and multifaceted. Participants provided rich descriptions of how psychosis intersected with their sense of self and their social identity, often bringing to light vulnerabilities that made them feel exposed. The findings reflect the deeply personal nature of many psychosis‐related experiences and illustrate how psychosis can disrupt participants' ability to present themselves in a way they consider acceptable or intentional, leading to feelings of exposure and shame. The themes across the data were: psychosis can expose underlying shame; psychosis can cause shame; efforts to minimise, avoid, and resist shame; and shame can be triggered in mental health services.

### 4.1 Psychosis Can Expose Underlying Shame

Psychosis was seen to acutely expose sustained experiences of shame. Participants expressed the ways that experiences of psychosis could expose feelings and parts of themselves that they usually try to hide. Participants described usually presenting a ‘strong’ or intentional version of themselves to the world and feeling that psychosis interrupted this in a way that they couldn't control, revealing their vulnerability. This sense of exposure was associated with feelings of shame. Shame was described as a feeling that their flaws or a version of their private selves had been presented to the public, leading to embarrassment and fears about how others may perceive or judge them. Shame in this context could also be anticipatory, arising from a sense that others may see or find out about their flaws, or that these may be made visible by psychosis.

I had difficulty looking people in the eyes and I thought people would see something in me that wasn't right, which there was… something in me that wasn't right.—P14

Participants described feeling that their experiences of psychosis were visible to others, or a sense that psychosis was a ‘public’ experience in a way that allowed them to be judged. Participants were concerned that their behaviour drew attention from others, and this could be shameful. This was particularly marked when experiences of psychosis that participants recounted included beliefs that people were watching them, following them or keeping information about them.

I'd feel like my most vulnerable moments were very public and so then I wouldn't return to [a local place] because I'd had a psychosis and [been] living near there at the time and being out at the cafes and markets and stuff in a very psychotic way… while it's not really visible to people, the way I relate is very different and so in that regard it is visible.—P6

Psychosis could also expose historical shame. Psychosis experiences could lead participants to relive memories from the past that had shame attached, for example, hearing voices containing put‐downs from the past. Participants also described ways that psychosis could expose parts of themselves they had tried to leave behind, for example, delusions of being followed by people whom they had wronged in the past. In this way, psychosis was seen to tap into shame.

psychosis targets your weakest … you know weakest erm… you know your biggest fears.—P5

Psychosis could also expose versions of themselves that participants found incongruent to their feelings of self‐worth. For instance, shame could arise from the juxtaposition of experiences of feeling famous, important or grandiose with descriptions of feeling ‘small’ or ‘unimportant’ in their lives when not experiencing psychosis.

And I wanted to people [to] be like, “Oh she's in Byron. She's not this small, insignificant girl. She's actually doing things.” But now that I look back, I'm like, that was really attention‐seeking and I think I feel sorry for myself.—P14

### 4.2 Psychosis Can Cause Shame

Some experiences of shame were directly linked to psychosis experiences. These included shame from behaviours or actions occurring as part of the psychosis, or shame from the loss of control, loss of connection to others, or loss of self.

A number of participants identified specific instances of shame arising from their actions during psychosis, particularly when this behaviour was incongruent with how they would usually present themselves to others. Examples of this included ‘out‐there’ behaviour, saying rude or offensive things or acting in uncharacteristic ways.

Usually in a lot of the psychosis, I had hypersexuality…. And this particular time it was pretty extreme. And then I think there is definitely shame associated with that. That is the one thing that I probably look back on and just feel not particularly good about.—P12

This kind of trigger for shame could also be more generalised and relate more to the impact of not being able to act as they usually would.

I felt kind of ashamed that I wasn't myself….—P14

Participants also described shame arising from the beliefs they held when they were experiencing psychosis, for example, believing they were someone they were not. Shame could arise as a consequence of no longer trusting their own judgement.

Well sometimes it makes you question … you know—do I know what I know? … Can I trust my own interpretation of what the events are… kind of thing.—P5

Feelings of shame were both caused and triggered by the loss of control experienced in psychosis. Participants described feeling that they did not have control of their minds or bodies during episodes of psychosis and that this made them feel shame about actual and potential events and actions.

And you know, I was looking at my neighbour… and I was thinking, “I really hope she doesn't know anything about this.” …And then you feel ashamed. You're like, “Oh, I should never have, you know, never have done that.” But then when you're in psychosis, you're not in control of anything.—P12

Shame appeared to be compounded over time for participants, with early life experiences of shame reflected in later psychosis experiences. This was also apparent indirectly across the interviews where, for example, people who had early experiences of shame associated with rejection later described anticipatory shame from potential further rejection as an adult. Participants at times drew links between current feelings of shame associated with psychosis events and past shame.

I think [current shame from psychosis has] probably got something to do with the abuse that I suffered when I was eleven…. I think a lot of those feelings come from that, I think that's where it's coming from.—P7

Shame arising from the experience of psychosis could be pervasive across people's lives, influencing how they see themselves and imagine other's see them. Shame could occur from a loss of social status, rejection, isolation, or loss of life plans, leading people to feel that they were being seen differently by family or friends and contributing to further loss of relationships and missed career opportunities. This sort of shame was woven into experiences of loss and grief about how they thought life could have been without psychosis, and through social comparisons to peers.

Sometimes I feel like God has entitled everyone to something and I'm not entitled to it. For some unknown reason that's how I feel. I see men and women; they've got a child, and they've got family. They're driving a car and got the degree and they've got a full‐time job and I don't have those things. I can't drive. I can't do this. I can't do that, and I feel like God loves me even less, or I've been chosen before I'm born and God doesn't like me, and I'm not entitled to a lot of things in life that most people are.—P2

Shame about psychosis could also be activated when reflecting on these experiences. In the interviews, participants occasionally described feeling shame which was managed in the moment and also followed up afterwards. However, as one participant described when reflecting on how it felt to talk about these experiences:

I feel like I've taken all my clothes off and run down the street….—P7

### 4.3 Efforts to Minimise, Avoid and Resist Shame

Participants described a range of ways in which they consciously and unconsciously responded to feelings of shame. These included efforts to avoid further shame, for instance by socially withdrawing or disconnecting from others, or by making themselves less visible or ‘smaller’. Additionally, participants reported ‘people‐pleasing’ in efforts to avoid further social exclusion or disapproval. People‐pleasing was also used when individuals anticipated that shame may arise in situations or interactions.

Participants described ways of actively seeking to reduce shame, particularly around mental illness. This was commonly through bids for connection with others that contributed to destigmatising experiences of psychosis. For instance, talking with others who had similar lived experiences through formal or informal networks, or by connecting with supportive professionals.

I think talking about something reduces your shame and your stigma, and reduces… how you feel… If you're feeling terrible about yourself, it can really help to chat to someone about it.—P12

Some participants sought to minimise shame by detaching from diagnostic labels such as ‘psychosis’ and finding alternative ways to describe their experiences that were meaningful to them, for example ‘being keyed in’ or experiencing ‘alternate realities’. These ways of understanding and talking about their experiences were used to reduce shame. Participants at times also minimised their own experiences by highlighting them as ‘different’ to psychosis experienced by others. For example, talking about how they were less affected by experiences of psychosis than others, or why the term didn't describe their experiences. This provided distance between stereotypes of psychosis and their own experiences, potentially to minimise shame.

You can tell when it's really broken someone down. Some of the people in rehab are completely broken down and they talk with a shaky high voice. Yeah, I feel bad for them.—P10

For other participants, experiences of psychosis were understood as a part of who they were but did not form their whole identity. This gave participants a way to distance themselves from the shame associated with and activated by psychosis, and to reconnect to their sense of self held prior to psychosis, or to adapt their identity to one in which psychosis was only one part.

[psychosis] completely disrupted all those thoughts and things so at the end of it I felt kind of hollow and really numb. But now I'm starting to feel a bit more like myself again and like I've got a place to live and all these little things that are starting to begin to make me feel a bit better, to remind me of who I am.—P11

Some participants described a resistance, or resilience, to shame in the context of psychosis. At times this was through seeking to understand the meaning behind psychosis experiences or through finding strength in what they had been through. Participants reflected that psychosis experiences helped them to grow, to become stronger and to gain a better understanding of themselves. This was particularly apparent amongst younger participants where ‘being different’ was framed as a positive, for example, that they have a better understanding of themselves, or have something that others do not. This could also occur over time and with a sense of stability.

Life is better now. It doesn't seem that way to think that it's better now after going through psychosis, but from who I was before, the psychosis has changed me as a person and it makes me stronger. It makes me want to work towards making the most of life and appreciating life. Whereas before I didn't really appreciate life as much as I do now.—P9

### 4.4 Shame Can Be Triggered in Mental Health Services

Participants talked extensively about experiences of shame which occurred within mental health services. These experiences included shame as a result of systemic factors such as the environments where care was delivered and the ways that services were structured and available, as well as in interactions with mental health professionals in any context. Experiences of shame in mental health services could damage trust in care providers and create barriers to seeking help. Some of these experiences were subtle, and some were more obvious.

… particularly when you go into emergency and they shackle you to the bed, I mean that really puts you off asking for help.—P6

Participants described the environment and structures where care is delivered as akin to being in prison. For instance, loss of freedoms, lack of structure or social opportunities, and policies that seemed punitive rather than recovery‐oriented. Participants noted that there were separations between themselves and staff who made them feel othered and shut out. These separations were both physical in the form of staff areas and locked doors, as well as implied through a lack of information sharing or the use of inaccessible language.

It's all medical terminology, and you can't absorb it and understand it. It's like another language, even though it's English. You can't understand it, and you're not at your best to read it.—P2

Participants described experiences that were reminiscent of shame associated with coercive practices such as use of restraint, sedation or forced medication, as well as a lack of control, agency or responsibility. Dehumanising practices were also described such as having to ‘fight for food’ or being talked about as if they were not present. Participants' descriptions of shame in services often mirrored previous experiences of shame or trauma. For example, some spoke of times in services which reenacted past experiences of a lack of agency, being coerced or being made to feel small and insignificant.

Yeah, particularly the shackling… I get quite distressed, it happens in the mornings, I'm reminded of what occurred and I feel quite ashamed and frightened and traumatised by it…it still lingers with me.—P6

The power imbalance between staff and people accessing care was both shaming and triggering of past experiences. Participants drew links between experiences in care and past experiences where they had also felt trapped, overpowered, or helpless.

Yeah, being trapped… sometimes being trapped in hospital, you know… you don't know what their intentions are. Sometimes it is distressing. You know… they have all the power.—P5

Participants noted that shame was rarely asked about in services and identified that it could be valuable to discuss and address it. Participants identified that shame could be minimised in interactions if mental health professionals sought to get to know people beyond their diagnoses and took the time to ensure that people had choices, understood their treatment plan and had their needs met. Positive experiences that counteracted shame were apparent in descriptions of interactions where participants felt ‘heard’, staff showed empathy, or they were given choices about care.

So sit down for a conversation rather than knee jerk reactions and ask me about what I'm experiencing, ask me about my life, ask me about who I am, and have some empathy towards how frightened I am.—P6

## 5 Discussion

These findings provide new and detailed knowledge about the relationship between shame and psychosis from the perspectives of people with lived experience. The findings indicated that psychosis can amplify shame and cause shame, with participants sharing the efforts that they had to take to minimise the impact of shame. Shame was also identified to be triggered and caused by factors generated within the context of mental health care. The findings highlight a complex interaction between shame and psychosis and the diverse sources from which shame may arise, with implications for assessing, addressing, and minimising shame in clinical settings. The findings contribute to a growing field of literature about the need for shame‐sensitive and trauma‐informed mental health services.

While some of the experiences participants shared are resonant to findings across the literature, there has been very limited qualitative exploration of the experience of shame in the context of psychosis. The findings are resonant to theoretical ideas of shame identified in clinical contexts and experiences of mental illness (e.g., Dolezal and Gibson 2022; Jaeb 2022; Davies et al. 2024) and emerging understandings of the potential for shame in psychiatric care (Crossley and Jones 2011). However, in listening to the experiences of people living with psychosis and shame, a deeper understanding of the complexity of the nature of the relationship of these experiences is possible. The findings show that shame arising from experiences of psychosis is pervasive, and that historical or underlying shame is also common and can be reactivated by psychosis experiences. Shame also appears to be cumulative, with people who experience early shame susceptible to reminiscent experiences of shame associated with psychosis in adulthood. This aligns with research which suggests that early shame experiences can act as a reference point that primes individuals to interpret future events through a shame lens (Pinto‐Gouveia and Matos 2011). Some researchers suggest this existing shame may contribute to the development of psychosis experiences through the adoption of absorbed and avoidant coping strategies (e.g., rumination, hypervigilance, dissociation and emotional suppression) which increase the likelihood of cognitive or perceptual intrusions (McCarthy‐Jones 2017). This is further supported by a recent study that found shame to partially mediate the relationship between past trauma and experiences of psychosis (Davies, Lappin, Briggs, et al. 2025). Given the diverse sources that contribute to feelings of shame, efforts to reduce shame may therefore need to go beyond attempts to address those associated with mental illness (e.g., reducing stigma) and additionally engage with a person's life story and the meaning of past events.

The findings highlight that psychosis can bring experiences of shame from past adverse experiences to the forefront, with participants identifying efforts to find meaning in or understand experiences of psychosis to reduce shame. Finding meaning in experiences of psychosis can provide individuals with more understanding about sources of shame (e.g., exploring why they might feel shame around specific thoughts or behaviours) and help inform the development of new narratives. This mirrors the approach outlined in the Power, Threat, Meaning Framework (Johnstone et al. 2018) (PTMF), in which the influence of life events on psychological wellbeing is considered as an alternative to traditional diagnoses. Within the PTMF, shame is considered one way of interpreting threats to the self and the meaning given to them in the context of the broader social environment. Within this framework, personal narratives play a central role in understanding past experiences, with the meaning applied shaping the type of responses a person may have. Incorporating narrative or meaning‐making approaches such as those that align with the PTMF principles when supporting individuals with experiences of psychosis and shame may be beneficial in supporting them to make sense of their experiences, relieving the effects of shame.

The findings have implications for trauma‐informed care (TIC) models. TIC is increasingly being incorporated into policy and practice across mental health services. Whilst TIC principles inherently aim to avoid provoking shame by recognising the traumatic origins of ‘reactive’ behaviours, there is value in TIC approaches more explicitly addressing shame. One way this could be achieved is through shame‐sensitive practice (Dolezal and Gibson 2022). Shame‐sensitive practice involves acknowledging and understanding shame (e.g., what it is, how it is triggered and the impacts it may have); avoiding shaming (e.g., being cognisant of power imbalances and managing them appropriately); and addressing shame through creating emotional safety and developing shame resilience (Dolezal and Gibson 2022). Understanding shame as a component of trauma may aid in consolidating aligned work in these fields. Future work is needed to explore the influence of adverse events on shame in more detail, for instance, how shame associated with past events continues to influence experiences of psychosis.

Beyond targeted interventions, the findings of this study demonstrate that there is a need for all clinicians and services that provide mental health support to reflect on ways to avoid inadvertently contributing to, amplifying or causing shame. This can occur through increased awareness of shame, reducing shaming practices, ensuring opportunities for processing experiences in care and through reviewing the language that is used to describe and talk about experiences. In line with the experiences of participants in this study, stigmatising labels and medical terminology are known to perpetuate feelings of shame (Dolezal and Lyons 2017). Adopting terms used by people with lived experience aids connection and reduces feelings of exclusion. In this study, for example, while the term ‘psychosis’ was required for ethics, recruitment and study definition, transparency and collaboration in language use throughout demonstrated respect for people's diverse experiences and understandings of these. Greater incorporation into mental health services of approaches that support people to define their own experiences may normalise experiences, foster connection and reduce shame.

## 6 Limitations

The experiences of participants in this study have occurred within specific care contexts and experiences that may not resonate with all individuals living with psychosis or accessing mental health care. Only participants already engaged in services and willing to talk about their experiences of shame participated. It is likely that many people have other experiences that they were not able or willing to share. Talking about shame is inherently uncomfortable, and it was difficult to talk about shame in the study without activating experiences of shame for participants. It is therefore possible that participants did not express all aspects of their experiences that linked to shame. Similarly, while this project strived to be conducted in a trauma‐informed and shame‐sensitive manner, it cannot be assumed that people felt safe, and it is acknowledged that many people have highly refined and effective ways of disguising shame.

## 7 Conclusion

The findings of this study provide important insights about the nature and importance of the relationship between shame and psychosis from the perspectives of people living with psychosis. The experiences outlined in the accounts of participants highlight that psychosis can both contribute to new experiences of shame and reactivate shame from past experiences, and that shame experiences can be replicated and amplified within current care contexts. Thus, mental health services should strive to be shame‐sensitive within trauma‐informed care.

## 8 Relevance for Clinical Practice

This study has direct relevance to mental health nurses who support people experiencing psychosis and other mental health challenges. The findings highlight that shame is common and can have a distressing impact, with mental health services and clinicians contributing to these experiences. Awareness of possible sources of shame within service contexts and consideration of how shame‐sensitivity can be applied by nurses will be helpful in addressing shame, particularly within models of trauma‐informed care.

## Author Contributions

All authors listed meet the authorship criteria according to the latest guidelines of the International Committee of Medical Journal Editors. All authors have read and agree with the final manuscript.

## Ethics Statement

Ethics approval was provided by the South Eastern Sydney Local Health District HREC (Ref: 2022/ETH02000).

## Consent

Consent for publication was obtained as part of informed consent for the study.

## Conflicts of Interest

The authors declare no conflicts of interest.

## Data Availability

Research data are not shared.

## References

[inm70070-bib-0001] Arciniegas, D. B. 2015. “Psychosis.” Continuum 3: 715–736. 10.1212/01.CON.0000466662.89908.e7 PMC4455840 26039850

[inm70070-bib-0002] Aunjitsakul, W. , N. McGuire , H. J. McLeod , and A. Gumley . 2021. “Candidate Factors Maintaining Social Anxiety in the Context of Psychotic Experiences: A Systematic Review.” Schizophrenia Bulletin 47, no. 5: 1218–1242. 33778868 10.1093/schbul/sbab026 PMC8379542

[inm70070-bib-0003] Braun, V. , and V. Clarke . 2021. “To Saturate or Not to Saturate? Questioning Data Saturation as a Useful Concept for Thematic Analysis and Sample‐Size Rationales.” Qualitative Research in Sport, Exercise and Health 13, no. 2: 201–216.

[inm70070-bib-0004] Braun, V. , and V. Clarke . 2022. Thematic Analysis: A Practical Guide. SAGE Publications Ltd.

[inm70070-bib-0005] Braun, V. , and V. Clarke . 2023. “Toward Good Practice in Thematic Analysis: Avoiding Common Problems and Be(Com)ing a Knowing Researcher.” International Journal of Transgender Health 24, no. 1: 1–6. 36713144 10.1080/26895269.2022.2129597 PMC9879167

[inm70070-bib-0006] Carden, L. J. , P. Saini , C. Seddon , M. Watkins , and P. J. Taylor . 2020. “Shame and the Psychosis Continuum: A Systematic Review of the Literature.” Psychology and Psychotherapy: Theory, Research and Practice 93, no. 1: 160–186. 10.1111/papt.12204 30426672

[inm70070-bib-0007] Castilho, P. , A. M. Pinto , R. Viegas , S. Carvalho , N. Madeira , and M. J. Martins . 2019. “External Shame as a Mediator Between Paranoia and Social Safeness in Psychosis.” Clinical Psychologist 23, no. 2: 144–151.

[inm70070-bib-0008] Connor, C. , and M. Birchwood . 2013. “Through the Looking Glass: Self‐Reassuring Meta‐Cognitive Capacity and Its Relationship With the Thematic Content of Voices.” Frontiers in Human Neuroscience 7: 213. 23734118 10.3389/fnhum.2013.00213 PMC3659289

[inm70070-bib-0009] Corstens, D. , and E. Longden . 2013. “The Origins of Voices: Links Between Life History and Voice Hearing in a Survey of 100 Cases.” Psychosis 5, no. 3: 270–285.

[inm70070-bib-0010] Crossley, D. R. , and A. C. Jones . 2011. “Shame and Acute Psychiatric In‐Patient Care.” Psychiatrist 35, no. 11: 408–412.

[inm70070-bib-0011] Davies, K. , J. Lappin , S. Isobel , et al. 2025. “Shame and Trauma Are Critical to Understanding the Impacts of Psychosis: An Audit of Clinical Data From a Tertiary Psychosis Service.” Manuscript in Preparation. 10.1177/00048674251411085 PMC13191054 41589323

[inm70070-bib-0013] Davies, K. , J. M. Lappin , C. Gott , et al. 2024. “Experiencing Psychosis and Shame: A Systematic Review and Meta‐Analysis of the Strength and Patterns of Association.” Schizophrenia Bulletin: sbae139. 10.1093/schbul/sbae139. 39175117 PMC12414562

[inm70070-bib-0012] Davies, K. , J. M. Lappin , N. Briggs , S. Isobel , and Z. Steel . 2025. “Does Shame Mediate the Influence of Trauma on Psychosis? A Systematic Review and Meta‐Analytic Structural Equation Modelling Approach.” Schizophrenia Research 275: 87–97. 39693680 10.1016/j.schres.2024.12.008

[inm70070-bib-0015] Dolezal, L. , and B. Lyons . 2017. “Health‐Related Shame: An Affective Determinant of Health?” Medical Humanities 43, no. 4: 257–263. 28596218 10.1136/medhum-2017-011186 PMC5739839

[inm70070-bib-0014] Dolezal, L. , and M. Gibson . 2022. “Beyond a Trauma‐Informed Approach and Towards Shame‐Sensitive Practice.” Humanities & Social Sciences Communications 9, no. 1: 214. 35791341 10.1057/s41599-022-01227-z PMC7612965

[inm70070-bib-0016] Gilbert, P. 2003. “Evolution, Social Roles, and the Differences in Shame and Guilt.” Social Research 70, no. 4: 1205–1230.

[inm70070-bib-0017] Isobel, S. 2021. “Trauma‐Informed Qualitative Research: Some Methodological and Practical Considerations.” International Journal of Mental Health Nursing 30, no. Suppl 1: 1456–1469. 34310829 10.1111/inm.12914

[inm70070-bib-0033] Jaeb, M. A. 2022. “Concept Analysis of Shame in Nursing.” International journal of mental health nursing 31, no. 2: 295–304. 10.1111/inm.12948. 34750954

[inm70070-bib-0018] Johnstone, L. , M. Mary Boyle , J. Cromby , et al. 2018. The Power Threat Meaning Framework: Overview. British Psychological Society.

[inm70070-bib-0019] Kallio, H. , A.‐M. Pietilä , M. Johnson , and M. Kangasniemi . 2016. “Systematic Methodological Review: Developing a Framework for a Qualitative Semi‐Structured Interview Guide.” Journal of Advanced Nursing 72, no. 12: 2954–2965. 27221824 10.1111/jan.13031

[inm70070-bib-0020] La Bash, H. , and A. Papa . 2014. “Shame and PTSD Symptoms.” Psychological Trauma Theory Research Practice and Policy 6, no. 2: 159–166.

[inm70070-bib-0021] Lingard, L. 2019. “Beyond the Default Colon: Effective Use of Quotes in Qualitative Research.” Perspectives on Medical Education 8, no. 6: 360–364. 31758490 10.1007/s40037-019-00550-7 PMC6904374

[inm70070-bib-0022] Maijer, K. , M. J. H. Begemann , S. J. M. C. Palmen , S. Leucht , and I. E. C. Sommer . 2018. “Auditory Hallucinations Across the Lifespan: A Systematic Review and Meta‐Analysis.” Psychological Medicine 48, no. 6: 879–888. 28956518 10.1017/S0033291717002367

[inm70070-bib-0023] McCarthy‐Jones, S. 2017. “Is Shame Hallucinogenic?” Frontiers in Psychology 8: 1310. 28824494 10.3389/fpsyg.2017.01310 PMC5541028

[inm70070-bib-0024] Neale, J. 2016. “Iterative Categorization (IC): A Systematic Technique for Analysing Qualitative Data.” Addiction 111, no. 6: 1096–1106. 26806155 10.1111/add.13314 PMC5069594

[inm70070-bib-0032] O'Brien, B. C. , I. B. Harris , T. J. Beckman , et al. 2014. “Standards for Reporting Qualitative Research: A Synthesis of Recommendations.” Academic medicine 89, no. 9: 1245–1251. 10.1097/ACM.0000000000000388. 24979285

[inm70070-bib-0025] Peters, E. , T. Ward , M. Jackson , et al. 2017. “Clinical Relevance of Appraisals of Persistent Psychotic Experiences in People With and Without a Need for Care: An Experimental Study.” Lancet Psychiatry 4, no. 12: 927–936. 29179936 10.1016/S2215-0366(17)30409-1 PMC5714590

[inm70070-bib-0026] Pinto‐Gouveia, J. , and M. Matos . 2011. “Can Shame Memories Become a Key to Identity? The Centrality of Shame Memories Predicts Psychopathology.” Applied Cognitive Psychology 25, no. 2: 281–290.

[inm70070-bib-0027] QSR International Pty Ltd . 2018. NVivo Qualitative Data Analysis. 12th ed.

[inm70070-bib-0028] Stebbins, R. A. 2001. Exploratory Research in the Social Sciences. Sage.

[inm70070-bib-0029] Turner, M. H. , M. Bernard , M. Birchwood , C. Jackson , and C. Jones . 2013. “The Contribution of Shame to Post‐Psychotic Trauma.” British Journal of Clinical Psychology 52, no. 2: 162–182. 24215146 10.1111/bjc.12007

[inm70070-bib-0030] Varese, F. , F. Smeets , M. Drukker , et al. 2012. “Childhood Adversities Increase the Risk of Psychosis: A Meta‐Analysis of Patient‐Control, Prospective‐ and Cross‐Sectional Cohort Studies.” Schizophrenia Bulletin 38, no. 4: 661–671. 22461484 10.1093/schbul/sbs050 PMC3406538

[inm70070-bib-0031] Wood, L. , and C. Irons . 2016. “Exploring the Associations Between Social Rank and External Shame With Experiences of Psychosis.” Behavioural and Cognitive Psychotherapy 44, no. 5: 527–538. 26400370 10.1017/S1352465815000570

